# Functional coronary assessment in angina with intermediate coronary stenosis: the #FullPhysiology approach

**DOI:** 10.1093/eurheartj/ehae926

**Published:** 2025-01-10

**Authors:** Antonio Maria Leone, Domenico Galante, Andrea Viceré, Andrea Marrone, Filippo Maria Verardi, Chiara Giuliana, Ciro Pollio Benvenuto, Vincenzo Viccaro, Simona Todisco, Andrea Erriquez, Simone Biscaglia, Cristina Aurigemma, Enrico Romagnoli, Rocco Antonio Montone, Michele Basile, Eugenio Di Brino, Filippo Rumi, Gennaro Capalbo, Carlo Trani, Francesco Burzotta, Filippo Crea, Italo Porto, Gianluca Campo

**Affiliations:** Center of Excellence of Cardiovascular Sciences, Ospedale Isola Tiberina—Gemelli Isola, Via di Ponte Quattro Capi 39, 00186 Rome, Italy; Università Cattolica del Sacro Cuore, Largo Agostino Gemelli 1, 00168 Rome, Italy; Center of Excellence of Cardiovascular Sciences, Ospedale Isola Tiberina—Gemelli Isola, Via di Ponte Quattro Capi 39, 00186 Rome, Italy; Università Cattolica del Sacro Cuore, Largo Agostino Gemelli 1, 00168 Rome, Italy; Cardiology Unit, Azienda Ospedaliero Universitaria di Ferrara, Via Aldo Moro 8, 44124 Cona, Italy; Cardiology Unit, Azienda Ospedaliero Universitaria di Ferrara, Via Aldo Moro 8, 44124 Cona, Italy; Università Cattolica del Sacro Cuore, Largo Agostino Gemelli 1, 00168 Rome, Italy; Università Cattolica del Sacro Cuore, Largo Agostino Gemelli 1, 00168 Rome, Italy; Università Cattolica del Sacro Cuore, Largo Agostino Gemelli 1, 00168 Rome, Italy; Università Cattolica del Sacro Cuore, Largo Agostino Gemelli 1, 00168 Rome, Italy; Cardiology Unit, Azienda Ospedaliero Universitaria di Ferrara, Via Aldo Moro 8, 44124 Cona, Italy; Cardiology Unit, Azienda Ospedaliero Universitaria di Ferrara, Via Aldo Moro 8, 44124 Cona, Italy; Department of Cardiovascular Sciences, Fondazione Policlinico Agostino Gemelli IRCCS, Largo Agostino Gemelli 1, 00168 Rome, Italy; Department of Cardiovascular Sciences, Fondazione Policlinico Agostino Gemelli IRCCS, Largo Agostino Gemelli 1, 00168 Rome, Italy; Department of Cardiovascular Sciences, Fondazione Policlinico Agostino Gemelli IRCCS, Largo Agostino Gemelli 1, 00168 Rome, Italy; Università Cattolica del Sacro Cuore, Largo Agostino Gemelli 1, 00168 Rome, Italy; Alta Scuola di Economia e Management dei Sistemi Sanitari, Università Cattolica del Sacro Cuore, Largo Agostino Gemelli 1, 00168 Rome, Italy; Università Cattolica del Sacro Cuore, Largo Agostino Gemelli 1, 00168 Rome, Italy; Alta Scuola di Economia e Management dei Sistemi Sanitari, Università Cattolica del Sacro Cuore, Largo Agostino Gemelli 1, 00168 Rome, Italy; Università Cattolica del Sacro Cuore, Largo Agostino Gemelli 1, 00168 Rome, Italy; Alta Scuola di Economia e Management dei Sistemi Sanitari, Università Cattolica del Sacro Cuore, Largo Agostino Gemelli 1, 00168 Rome, Italy; Center of Excellence of Cardiovascular Sciences, Ospedale Isola Tiberina—Gemelli Isola, Via di Ponte Quattro Capi 39, 00186 Rome, Italy; Università Cattolica del Sacro Cuore, Largo Agostino Gemelli 1, 00168 Rome, Italy; Department of Cardiovascular Sciences, Fondazione Policlinico Agostino Gemelli IRCCS, Largo Agostino Gemelli 1, 00168 Rome, Italy; Università Cattolica del Sacro Cuore, Largo Agostino Gemelli 1, 00168 Rome, Italy; Department of Cardiovascular Sciences, Fondazione Policlinico Agostino Gemelli IRCCS, Largo Agostino Gemelli 1, 00168 Rome, Italy; Center of Excellence of Cardiovascular Sciences, Ospedale Isola Tiberina—Gemelli Isola, Via di Ponte Quattro Capi 39, 00186 Rome, Italy; Università Cattolica del Sacro Cuore, Largo Agostino Gemelli 1, 00168 Rome, Italy; Cardiovascular Disease Unit, IRCCS Policlinic Hospital San Martino, IRCCS Italian Cardiovascular Network, Largo Rosanna Benzi 10, 16132 Genoa, Italy; Department of Internal Medicine, University of Genoa, Largo Rosanna Benzi 10, 16132 Genoa, Italy; Cardiology Unit, Azienda Ospedaliero Universitaria di Ferrara, Via Aldo Moro 8, 44124 Cona, Italy

**Keywords:** Angina with non-obstructive coronary artery disease (ANOCA), Coronary flow reserve (CFR), Index of microcirculatory resistance (IMR), Coronary Vasomotor Disorders International Study (COVADIS), #FullPhysiology (#FP)

## Introduction

Recent studies and guidelines support the role of a comprehensive invasive functional approach in patients showing angina with non-obstructive coronary artery disease.^1,2^ This strategy allows identifying the correct endotype and tailoring medical therapy to symptoms and quality of life.^3^ In this regard, we recently proposed the #FullPhysiology (#FP) algorithm,^4^ which recommends the measurement of the coronary flow reserve (CFR) and index of microcirculatory resistance (IMR) using the bolus-thermodilution technique and the vasoreactivity testing with acetylcholine in accordance with the COVADIS (Coronary Vasomotor Disorders International Study Group) criteria^2^ on top of a conventional epicardial assessment. However, the additional value of the invasive functional tests in patients with epicardial intermediate stenosis judged non-obstructive at the conventional physiological assessment in clinical practice is less established. To fill this gap, the effects on clinical and economic outcomes of a treatment guided by a comprehensive #FP algorithm^4^ was compared to those of a conventional invasive epicardial assessment in patients with intermediate stenosis.

## Methods

The Fondazione Policlinico Universitario Agostino Gemelli and the Sant’Anna university hospitals in Rome and Ferrara have ongoing prospective registries [Post-Revascularization Optimization and PHysiological Evaluation of intermediaTe Lesions (PROPHET-FFR, NCT05056662); Prospective Registry of Acute Coronary Syndromes in Ferrara (ARYOSTO: NCT02438085); The Clinical Outcome of FFR-guided Revascularization Strategy of Coronary Lesions (HALE-BOPP: NCT03079739)] including all patients undergoing physiological assessment for chronic or stabilized acute coronary syndromes.^5–7^ Starting from these databases, we extracted patients meeting the following inclusion criteria: (i) chronic coronary syndromes exhibiting angina severity Canadian Cardiovascular Society (CCS) class ≥1 and/or evidence of myocardial ischaemia at non-invasive testing, (ii) a physiologically non-significant single-vessel disease [defined as a single lesion having %DS 40%–70% on visual estimation and a fractional flow reserve (FFR) > 0.80]. Exclusion criteria were acute clinical presentation or multi-vessel disease requiring revascularization in other vessels. Then, the study population was allocated to two consecutive cohorts according to the date of implementation of the #FP approach. The #FP interventional cohort included patients undergoing a comprehensive physiological assessment enrolled from 2020 to May 2023. The outcomes were compared to those of an historical control cohort including patients undergoing only conventional physiological assessment of the epicardial disease by FFR [conventional physiology (CP)] enrolled from 2015 to 2019. In the #FP cohort, patients were allocated to four groups and treated medically accordingly^4^: (i) coronary microvascular dysfunction (CMD; CFR <2.0 and/or IMR ≥25) treated with beta-blockers ± ranolazine; (ii) vasospastic disorders (epicardial spasm and/or microvascular spasm) according to the COVADIS criteria^4^ treated with calcium channel blockers; (iii) mixed endotypes treated with a combination of the previous therapies according to the most prevalent endotype; and (iv) normal #FP treated only with secondary prevention. The primary endpoint was the combination of the 1-year occurrence of cardiac death, myocardial infarction, target vessel revascularization (TVR), and rehospitalization for cardiac causes (including unstable angina, heart failure, and cardiac arrhythmias). Secondary endpoints included the individual components of the primary endpoint, the symptoms defined according to CCS classification, the number of non-invasive ischaemic tests performed during the follow-up period, and the healthcare costs according to the payer’s perspective by applying reimbursement tariffs.^8^ All the analyses were replicated in a matched population accounting for all baseline characteristics that significantly differed between the two groups using a 1:1 nearest neighbour propensity score matching without replacement. A logistic regression model was used to calculate the propensity score employing a matching distance of <0.1 as matching criterion.

## Results

**Figure 1:**
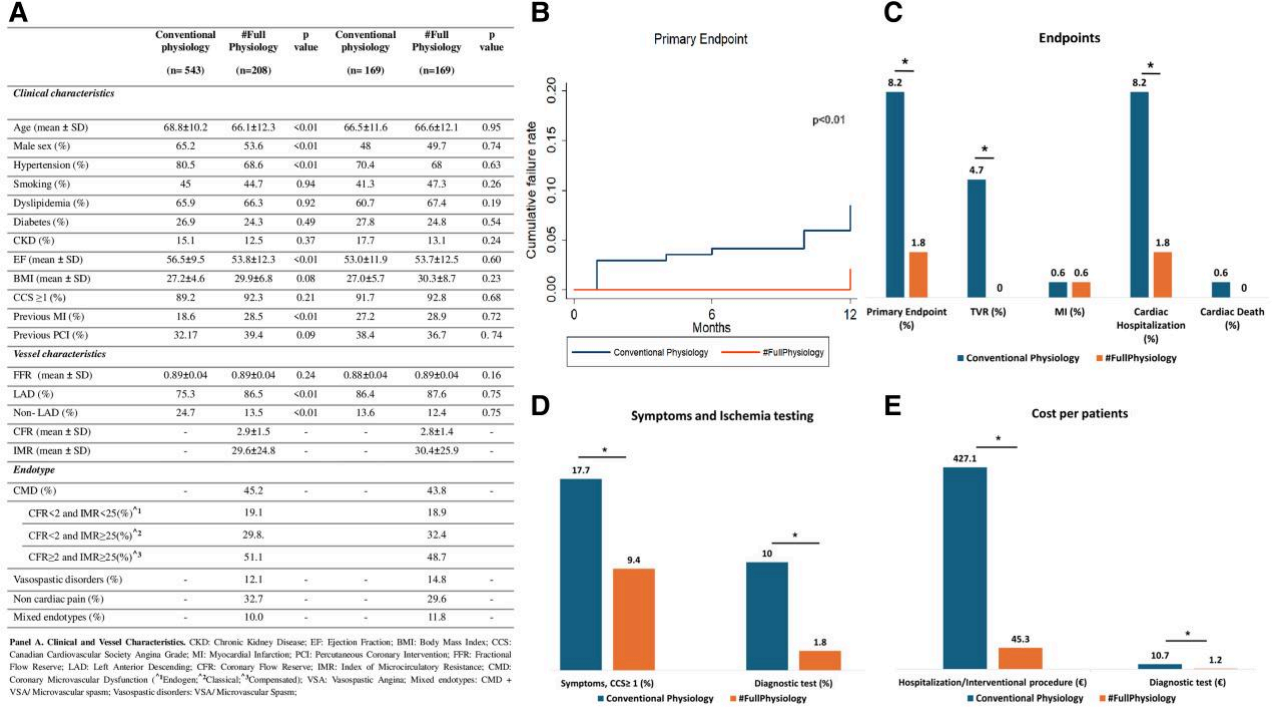
Baseline characteristics and clinical and economic outcomes in the study populations. (*A*) Clinical characteristics of patient populations. (*B*) Kaplan–Meier curves for the primary endpoint in the matched population. (*C*) Prevalence of the primary and secondary endpoint (myocardial infarction, target vessel revascularization, cardiac hospitalization, and cardiac death) in the matched population. (*D*) Proportion of symptomatic patients and the use of ischaemic diagnostic tests at follow-up across the matched population. (*E*) Economic outcomes in the matched population. MI, myocardial infarction; TVR, target vessel revascularization; CCS, Canadian Cardiovascular Society Angina Grade

A total of 751 patients (543 in the CP and 208 in the #FP group) were included (*Figure 1A*). Patients in the CP group were older with a higher prevalence of male sex and hypertension. Patients in the #FP group exhibited lower values of ejection fraction and a higher prevalence of previous myocardial infarction. Prevalence and severity of symptoms as well as baseline FFR was similar in the two groups. The prevalence of Groups 1, 2, 3, and 4 was 45.2%, 12.1%, 32.7%, and 10%, respectively. Patients undergoing #FP had a lower rate of the primary endpoint at 1-year follow-up (1.4% vs. 5.9%, *P* = .01), primarily driven by a reduction in TVR (0% vs. 2.6%, *P* = .02) and rehospitalizations (1.4% vs. 5.7%, *P* = .01) as compared to patients undergoing CP. In addition, patients undergoing #FP exhibited a significant reduction in anginal symptoms (CCS ≥1, 8.0% vs. 19.1%, *P* < .01) and recurrent ischaemia tests (1.4% vs. 9.5%, *P* < .01). These results were confirmed in a propensity score matched population of 169 CP and 169 #FP patients with similar clinical characteristics (age, sex, hypertension, ejection fraction, previous myocardial infarction, left anterior descending artery location, and diabetes). The rate of the primary endpoint was confirmed to be lower in the #FP group compared to the CP group, associated with a decreased prevalence of symptomatic patients and a lower rate of ischaemia tests (*Figure 1B–D*). Notably, the clinical advantage observed in the #FP group translated also into a substantial reduction in healthcare costs for both the overall population (cost of hospitalization/interventional procedures 36.8 ± 21.1€/patient in #FP vs. 263.2 ± 51.5€/patient in CP, *P* < .01; cost of new diagnostic tests 0.9 ± 0.5€/patient in #FP vs. 11.5 ± 1.9€/patient in CP, *P* < .01) and the matched population (*Figure 1E*).

## Discussion

Our findings support the notion that in anginal patients with CCS and intermediate stenoses, the implementation of a #FP approach may improve clinical outcomes and reduce healthcare costs in comparison to a conventional approach. Three important limitations of the current study are (i) the lack of randomization; (ii) the lack of assessment of vasoconstriction at the site of intermediate stenoses, which could be an additional cause of transient myocardial ischaemia in this patient population^9^; and (iii) the pooling of patients with CMD, CFR <2.0, and/or IMR ≥25 in the same therapeutic group while they might need different treatments.^10^ Thus, a more accurate characterization of the mechanisms of ischaemia in this patient population using the #FP approach might further improve the positive results observed in this study on clinical outcomes and healthcare costs.

## Declarations

### Data Availability

Data are available upon reasonable request to the corresponding author (Antonio Maria Leone, e-mail: antoniomarialeone@gmail.com; antoniomaria.leone@unicatt.it; antoniomaria.leone@fbf-isola.it).

### Pre-registered Clinical Trial Number

PROPHET-FFR: NCT05056662. ARYOSTO: NCT02438085. HALE-BOPP:NCT03079739.
